# Unconsciousness After Regional Anesthesia for Arteriovenous Fistula Revision in a Patient with End-stage Renal Disease

**DOI:** 10.7759/cureus.5045

**Published:** 2019-06-30

**Authors:** Sina Ghaffari, Nathalie Abitbol, Joni M Maga

**Affiliations:** 1 Anesthesiology, University of Miami, Miami, USA

**Keywords:** supraclavicular block, neuraxial anesthesia, complication of regional anesthesia

## Abstract

Chronic kidney disease can lead to different chronic complications. We describe a case where a patient with end-stage renal disease (ESRD) became unresponsive during transportation to the recovery room, following finishing an arteriovenous fistula revision. The patient had received supraclavicular block ninety minutes prior to the incident and surgery was finished under monitored anesthesia care (MAC). After five minutes of chest compression and intubation, monitoring showed normal sinus rhythm and the return of spontaneous circulation. The patient was transferred to the intensive care unit and extubated two days later while she was alert, oriented and hemodynamically stable. CT pulmonary angiogram showed no evidence of pulmonary emboli and echocardiogram did not show any further cardiac event comparing to preoperative status. Ruling out other differential diagnoses for patient’s unconsciousness, we have discussed the possibility of neuraxial anesthesia after upper extremity block in this patient.

## Introduction

Opening an arteriovenous fistula for dialysis is a very common procedure in patients with end-stage renal disease (ESRD). Different anesthesia techniques including general, regional, and local anesthesia with monitoring have been used for this procedure but upper extremity blocks including axillary, supraclavicular, and interscalene blocks are most commonly being used. 

The chronic renal disease can be accompanied by serious cardiac complications including coronary artery disease, hypertensive or ischemic cardiomyopathy resulting in pulmonary hypertension. The cardiorespiratory arrest has been reported in sporadic cases of AV fistula formation under different techniques of anesthesia, but this is the first report of neuraxial anesthesia following the ultrasound-guided supraclavicular block for this procedure. It is important that anesthesiologists who deal with these patients on a regular basis be aware of potential complications in these procedures.

## Case presentation

A 41-year-old female with a prolonged history of chronic kidney disease due to diabetes mellitus (DM) type I and failed kidney and pancreas transplant who was on hemodialysis three days a week scheduled for excision of left upper extremity arteriovenous graft with vein patch and arm bypass. The preoperative evaluation was consistent with DM, hypothyroidism, hypertension, coronary artery disease treated with CABG, and coronary stent placement. ECG showed left atrial enlargement and possible lateral wall ischemia. Transthoracic echocardiography showed mild concentric left ventricular hypertrophy with EF+60%. E/E’>20 cm/sec was indicative of elevated left ventricular end diastolic pressure. Right ventricular systolic pressure was elevated at 70-80 mmHg. Electrolytes were normal before surgery.

Because of preoperative cardiac histories and findings, regional anesthesia with monitoring and sedation during surgery was selected as the preferred anesthesia technique. Regional anesthesia team selected supraclavicular block considering the site of the surgery, which was high medial part of the arm, somehow near the axillary area. Block was placed in the preoperative holding area at the bedside and the patient was transferred to the operating room. Monitoring and sedation with Propofol 50-75 mic/kg/min were started. She also received 100 mics of fentanyl in two incremental doses. Patient’s hypertension was corrected after starting sedation. Other vital signs were stable, and she was comfortable and communicative for the most part of the surgery.

Surgery was finished after one hour and the patient was verbally communicative with anesthesia provider. While monitoring was unhooked, and she was ready for transferring on the recovery bed, she reported an electrical feeling in the whole body and became unresponsive. The patient was coded, and chest compression was established simultaneously with mask ventilation. The first ECG rhythm after establishing the monitors again was sinus but since she was unconscious, reanimation and resuscitation were continued with endotracheal tube intubation, IV fluid administration, and norepinephrine infusion. The patient was transferred to ICU to continue the supportive measures. All the metabolic reasons for unconsciousness were ruled out. CT angiography of the chest and brain CT scan was done to rule out pulmonary emboli or any intracranial source of unconsciousness which were both negative. Supportive measured continued until patient gained full consciousness two days later and extubation was done.

## Discussion

The prevalence of hemodialysis is increasing globally. There is an opinion that permanent vascular access is the preferred method for hemodialysis. On the other hand, patients with end-stage renal disease carry a high risk for cardiovascular comorbidity. Then, regional anesthesia is believed to be the superior technique, even better than local anesthesia, for arteriovenous shunt formation procedure. A study by Aitken and his colleagues showed that the brachial plexus block significantly improved three months of primary patency rates for arteriovenous fistula in upper limbs [1-2]. Although it is the safest method of anesthesia for these patients, respiratory complications like pneumothorax and phrenic nerve paralysis have been reported [3-4]. Other reported side effects of local anesthetics such as cardiac and central nervous system toxicity have been reported but they are less common with ropivacaine or prilocaine, which have been used in our patient [5].

In any circumstances of practicing with local anesthetics, knowledge of the pharmacokinetics, risk factors, prevention, and treatment of local anesthetic systemic toxicity is essential. Local anesthetic systemic toxicity usually presents with central nervous system excitation followed by inhibition. At the same time, it is followed by cardiovascular compromise due to arrhythmia, decreased cardiac contractility, and low systemic vascular resistance. Clinical presentation can be highly variable; therefore, atypical presentations are not uncommon [6].

Our case might have experienced an atypical local anesthetic systemic toxicity, because we neither observed any seizure activity nor arrhythmia before unconsciousness, but regarding the late onset of incident, the other more possible explanation is gradual spreading of the local anesthetics towards the epidural, subdural or intrathecal space, while pons and medulla will be anesthetized causing respiratory arrest and hypotension. Although it rarely happens, spreading of the local anesthetic into intrathecal, subdural or epidural space has been described after inter scalene block [7]. Though, this complication has never been reported with the ultrasound-guided supraclavicular block. The supraclavicular block was done in our patient with 30 ml of the mixture of Ropivacaine 0.5% and Mepivacaine 1.5%. The event happened 90 minutes after starting the block placement when surgery was finished and she was ready to be transferred on to the recovery bed, awake and verbally communicative with the anesthesia provider. Just before the incident, the patient complained of electrical activity in her whole body which can be explained by the possible cervical neuraxial distribution of local anesthetic. The gradual migration of the local anesthetic through the brachial plexus sheet can describe the late onset of this adverse event in our patient. Similar to the subdural spread of local anesthetic, this event also presented itself with unconsciousness, respiratory arrest, bradycardia, and hypotension [8]. On the contrary to cardiac toxicity of local anesthetics, patients are more responsive to treatment, which is ventilatory support, ionotropic medications, and fluid administration until the disappearance of the local anesthetic effect.

## Conclusions

The authors of this article have presented a possible rare complication of the supraclavicular block. The complications of the local anesthetics in regional anesthesia can be frequent and sometimes atypical. Prompt diagnosis of each type of complications depends on its clinical manifestation and treatment can be different, though cardiorespiratory supportive measures are always in priority. 
